# Bioimpedance Analysis of Cucumber Plants Exposed to Different Nitrogen Doses Under Greenhouse Conditions

**DOI:** 10.3390/s25082486

**Published:** 2025-04-15

**Authors:** Flórián Kovács, Katalin Juhos, Zoltán Vizvári, Péter Odry, Ingrid M. Gyalai, Peter Sarcevic, Ákos Odry

**Affiliations:** 1 Department of Agro-Environmental Studies, Hungarian University of Agriculture and Life Sciences, Villányi Str. 29–43, H-1118 Budapest, Hungary; kovacs.florian@szte.hu; 2 Institute of Plant Sciences and Environmental Protection, Faculty of Agriculture, University of Szeged, H-6800 Hódmezővásárhely, Hungary; gyalai.ingrid.melinda@szte.hu; 3 Biomatics and Applied Artificial Intelligence Institute, John von Neumann Faculty of Informatics, Óbuda University, Bécsi Str. 96, H-1034 Budapest, Hungary; vizvari.zoltan@nik.uni-obuda.hu; 4 Bioimpedance Technologies Research Center, University Research and Innovation Center, Óbuda University, Bécsi Str. 96, H-1034 Budapest, Hungary; 5 Multidisciplinary Medical and Engineering Cellular Bioimpedance Research Group, Szentagothai Research Centre, University of Pecs, Ifjusag Str. 20, H-7624 Pecs, Hungary; 6 Department of Control Engineering and Information Technology, University of Dunaújváros, Táncsics Mihály u. 1, H-2400 Dunaújváros, Hungary; podry@uniduna.hu (P.O.); odrya@mk.u-szeged.hu (Á.O.); 7 Department of Mechatronics and Automation, Faculty of Engineering, University of Szeged, Moszkvai Krt. 9, H-6725 Szeged, Hungary

**Keywords:** nitrogen monitoring, extracellular fluid resistance, sensitive Cole–Cole parameters, double-shell model, PSO

## Abstract

Nitrogen (N) availability is critical for cucumber (*Cucumis sativus* L.) growth and yield in greenhouse production. In this study, we investigated the effects of different N doses on the bioimpedance spectroscopy (BIS) parameters of cucumber plants (ES.22.17 F1 genotype), focusing on extracellular fluid resistance ($R_{1}$), intracellular fluid resistance ($R_{2}$), vacuole fluid resistance ($R_{4}$), and cell membrane capacitances ($C_{m}$, $C_{t}$). The results showed that low N supply significantly increased $R_{1}\;$and reduced $C_{m}$ in the leaves, indicative of decreased nitrate (NO_3_^−^) concentration and impaired membrane fluidity. Higher N supply lowered resistance and increased cell membrane capacitance, reflecting improved ion transport and storage efficiency. A strong positive correlation was observed between total N and NO_3_^−^ content (r = 0.9), while NO_3_^−^ content negatively correlated with extracellular fluid resistance ($R_{1}$, r = −0.8) and vacuole fluid resistance ($R_{4}$, r = −0.9). The optimal N supply for cucumber plants was associated with $R_{1}$ values of 47,121.07–52,953.93 Ω, $R_{4}$ values of 0.348–0.529 Ω, and $C_{m}$ values of 3.149 × 10⁻^10^–3.781 × 10⁻^10^ F. These BIS parameters showed high sensitivity to plant N status, highlighting BIS as a promising, minimally invasive technique for real-time nutrient monitoring. By integrating BIS data and horticultural best practices, growers can refine N fertilization strategies for better resource efficiency and potentially higher yields and fruit quality.

## 1. Introduction

Nitrogen (N) is a key nutrient for plant growth, essential for amino acids, proteins, cell structures, and chlorophyll synthesis [1]. The main forms of N used by plants are ammonium (NH_4_^+^) and nitrate (NO_3_^−^), with nitrate being particularly abundant [2]. Optimal N supply enhances chlorophyll synthesis and leaf longevity, which are crucial for biomass accumulation [3].

Cucumber (*Cucumis sativus* L.), one of the most significant greenhouse crops, is highly responsive to N management, which strongly influences both yield and fruit quality [4]. With rising artificial fertilizer prices, research into physiological factors affecting N fertilization efficiency is increasingly relevant. Cutting-edge, non-destructive diagnostic tools, such as hyperspectral imaging (HSI) and fluorescence-based sensors, have significantly improved the precision of N management. HSI enables the accurate prediction of leaf N content through spectral reflectance analysis [5], while fluorescence sensors, like the Multiplex^®^, provide indicators such as the Nitrogen Balance Index (NBI), which correlates with the Nitrogen Nutrition Index (NNI) to identify N deficiencies and optimize fertilization strategies [6].

Although traditional chemical N analysis methods are precise, they are time-consuming and not suitable for real-time monitoring. Recent advances in non-destructive approaches, based on spectral reflectance and chlorophyll estimation, allow for faster and periodic measurements across plants [7]. However, these methods face challenges related to environmental sensitivity and external confounding factors [8]. To address these limitations, integrating advanced diagnostic tools with mathematical models that account for the physical and chemical interactions in plant tissues could further enhance nutrient management in cucumber cultivation.

Among non-destructive monitoring methods, measuring bioimpedance spectroscopy (BIS) parameters also allows for rapid diagnostics, and it is widely used to evaluate biological tissues. In the BIS method, the frequency dependence of the impedance can be analyzed by performing a cell-based electrical circuit model analysis [9]. One of the primary advantages of the BIS method is its reduced sensitivity to external light conditions, enabling more stable measurements. Additionally, its relatively low data memory requirements facilitate real-time monitoring [10]. However, water deficiency and other environmental factors can still influence BIS readings [11]. Therefore, the careful standardization of conditions such as irrigation and temperature is essential for obtaining reliable measurements [11,12,13].

Some studies have already demonstrated the potential of BIS to detect N deficiency symptoms in plants [1,14,15], as well as phosphorus (P) [16] and potassium (K) deficiency symptoms [17]. The results of [14] showed that BIS can be used for the early diagnosis and monitoring of tomato N nutrition stress. Furthermore, [15] observed a positive correlation between the N content of the plant and the frequency values of the minimum phase angle. Their results suggest that BIS could be sensitive to the N status of plants. Nevertheless, there is still little research on the study of plant nutrient status with a particular focus on the potential of BIS [18].

Previous research [14,15] has demonstrated that N significantly influences extracellular resistance (apoplastic fluid resistance), intracellular resistance (cytoplasmic fluid resistance), and cell membrane capacitance. However, these studies utilized the single Cole–Cole model, which does not account for the vacuole’s capacitance and resistance. Considering that NO_3_^−^ is primarily stored in the vacuole, we found it appropriate to conduct a comprehensive analysis using the Double-Shell model, which includes both vacuole capacitance and resistance. We assumed that this approach would provide a more complete understanding of N metabolism processes using BIS.

The primary objective of this study is to identify how different BIS parameters, including extracellular fluid resistance, intracellular fluid resistance, vacuole fluid resistance, cell membrane capacitance, and vacuole capacitance, respond to different levels of N supply in cucumber plants under greenhouse conditions. We are trying to determine whether these measurements can provide insights into the determination of optimal N levels for specific cropping practices, thus contributing to more accurate and sustainable N management strategies for cucumber production.

The primary contributions of the study are as follows:

(1) We show that both resistance (extracellular fluid resistance, intercellular fluid resistance, and vacuole fluid resistance) and cell membrane capacitance values exhibit distinct changes with different N doses, indicating that even small changes in the NO_3_^−^ and total N content of leaves can be rapidly detected using minimally invasive BIS parameters.
(2) By correlating BIS-derived parameters with total N and NO_3_^−^ concentrations, we establish specific threshold intervals that robustly distinguish suboptimal from near-optimal N availability in cucumber leaves.
(3) Our results confirm that BIS can serve as a rapid, complementary diagnostic method alongside conventional chemical analysis, allowing timely adjustments to fertilization strategies, preventing overfertilization, and promoting more efficient N use in greenhouse cucumber production.

## 2. Materials and Methods

### 2.1. Experimental Setup and Treatments

The greenhouse experiment was conducted on cucumber plants (*Cucumis sativus* L.) of the ES2217 F1 genotype (Orosco Ltd., Orosco Kft., Orosháza, Hungary) with five replicates, involving a total of 20 plants. Seeds were sown in rockwool cube and irrigated with distilled water until the two-cotyledon phase. At the two-leaf stage, the plants were transferred to the greenhouse. During the experiment, the maximum daytime photosynthetic photon flux density (PPFD) in the greenhouse was 1520.9 μmol m^−2^ s^−1^ (average PPFD: 280.8 μmol m^−2^ s^−1^), daytime temperatures ranged between 16 and 33 °C (average: 28 °C), and nighttime temperatures never fell below 14 °C. The daytime relative humidity ranged from 30 to 93.1% (average: 63.8%).

Nutrient supply for the cucumber plants began immediately after transplanting. The nutrient solution was prepared using Solinure 5 General Purpose 20-20-20+TE fertilizer by dissolving 1.5 g of fertilizer in 1 L of water, resulting in the following millimolar concentrations: N-NO_3_: 1.04 mM; N-NH_4_: 2.83 mM; N-CO(NH_2_)_2_: 3.07 mM; P_2_O_5_: 2.11 mM; K_2_O: 3.18 mM; Fe: 10.7 µM; Mn: 2.7 µM; Br: 13.9 µM; Cu: 0.47 µM; Mo: 0.31 µM; Zn: 0.46 mM. The total N concentration (6.94 mM) is derived from the sum of the three N forms present in the fertilizer: nitrate (N-NO_3_: 1.04 mM), ammonium (N-NH_4_: 2.83 mM), and urea (N-CO(NH_2_)_2_: 3.07 mM). Based on a rough conversion (1 mM N ≈ 14 mg N per L), this standard solution provides a total of approximately 6.94 mM N, corresponding to ~97 mg N per L.

In greenhouse cucumber production, the recommended total N concentration in nutrient solutions commonly ranges between 100 and 200 mg N per L (depending on the growing medium and plant development stage). Thus, our initial fertilizer regime is near the lower boundary of generally recommended N supply rates.

After applying the standard fertilizer, increased N doses were introduced, starting from the stage when 2–3 true leaves had developed. The N concentration was increased using KNO_3_ at the following levels: N1: 1.64 mM; N2: 3.29 mM; N3: 4.92 mM; N4: 6.56 mM. These doses represent progressive increases in total available N, moving from a relatively low to a moderate/high N range compared to standard horticultural recommendations. Hence, the treatments allow us to assess cucumber responses to suboptimal, near-optimal, and potentially higher-than-usual N supply levels. During irrigation, the amount of nutrient solution provided per plant was initially 70–80 mL per irrigation, which was later increased to 150–200 mL per plant per irrigation, ensuring sufficient water and nutrient availability as the plants grew larger.

### 2.2. Plant Physiological Measurements

Physiological parameters were measured on cucumber plants at the 6–7 true leaf phenological stage. After each BIS measurement, the leaves were cut and submitted to plant physiological analysis. The methods, replication numbers, and phenological state for these measurements are summarized in Table 1.

The method described by [19] was employed to determine the concentrations of carotenoids and chlorophylls. The chlorophyll measurements include total chlorophyll content (total-chl), carotenoid content (carotenoids), chlorophyll-a content (chl-a), and chlorophyll-b content (chl-b). Pigment concentrations are expressed in mg g^−1^ fresh weight (FW).

Leaf chlorophyll content was assessed non-destructively using an Apogee MC-100 chlorophyll meter (Apogee Instruments, Inc., Logan, UT, USA). The MC-100 estimates chlorophyll concentration in µmol per cm^2^ of leaf area. The measurements were carried out following the methodology outlined by [20]. 

Cucumber leaf nitrate (NO_3_^−^) content was analyzed using the salicylic acid–sulfuric acid method, as described by [21] The NO_3_^−^ content of the plants was calculated by multiplying the NO_3_^−^ concentration in the leaves (mg g^−^¹ FW) by the fresh weight of the plant.

The total N content in cucumber leaves was determined using the Kjeldahl method, as outlined by [22]. A 0.25 g sample of ground leaf tissue was digested with 5 mL of concentrated H_2_SO_4_ at 380 °C, using Na_2_SO_4_ and selenium as catalysts, until the solution became clear. The digested sample was then diluted to a total volume of 50 mL with distilled water. A 10 mL aliquot of the diluted solution was distilled after adding 1 mL of 40% NaOH. The distillate was titrated with a 0.02 N H_2_SO_4_ standard solution until the endpoint was reached.

**Table 1:** Summary of methods and replication for plant physiological parameters.

Determination of Plant Physiology Characteristics			
Plant Parameter	Methods	Replication	Growth Stage
Leaf total N content (%)	[22]	4	
Leaf NO_3_^−^ content (mg g^−1^ FW)	[21]	4	6–7 true leaf stage of cucumber (ES2217 F1 genotype)
Photosynthetic pigments (mg g^−1^ FW)	[19]	4	
Chlorophyll Content Index (CCI, dimensionless unit) (Apogee MC-100)	[6,23]	4	

### 2.3. Bioimpedance Spectroscopy Measurements and Plant Cell Model

Bioimpedance (BIS) measurements were performed on cucumber leaves using a four-channel battery-powered impedance measuring instrument. This instrument, which has been described in earlier studies, was successfully utilized for measuring water stress in pepper plants [24]. Since the instrument was previously described in detail, only the key information is given; the interested reader is referred to [24].

Our four-channel instrument utilizes a digital lock-in amplifier, which precisely determines signal amplitude and phase at the reference frequency, even in low signal-to-noise conditions. The instrument supports both BIS and discrete frequency bioimpedance measurements. In BIS mode, users select frequency decades and points per decade. The built-in signal generator generates a monochromatic sine wave, and via the utilized electrodes measures the system response. The instrument applies a digital lock-in algorithm to compute the amplitude and phase results. The key characteristics of the instrument are specified in [24].

The instrument is utilized for biological process evaluation in plants using gold-coated needle electrodes. A frequency sweep from 1 Hz to 100 kHz, with ten points per decade, was performed for impedance data acquisition; then, the plant physiological parameters were extracted with numerical optimization.

The excitation signals used in the measurements were attenuated by 25 dB. The electrodes were connected to the bioimpedance analyzer via coaxial cables configured in a four-terminal pair arrangement.

**Figure 1:**
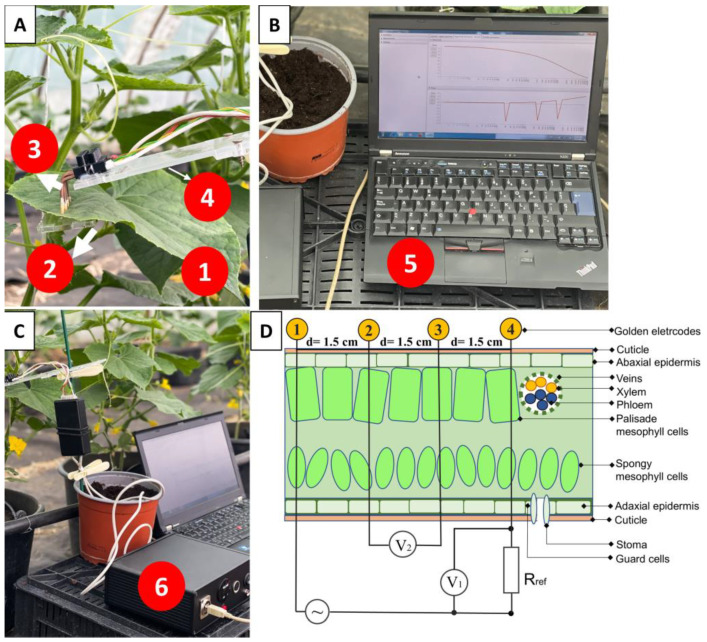
Schematic representation of the BIS instrument. (**A**) Position of electrodes used on cucumber plants: 1. Model plant (cucumber ES2217 F1 genotype); 2. Plastic to hold the electrodes; 3. Four golden electrodes; 4. Palette where the four gold electrodes are clamped. (**B**) 5. Measurement data displayed in a graphical interface. (**C**) 6. Measuring instrument (BIS). (**D**) Electrode arrangement within the leaf cross-section and the principle of BIS measurement according to [24].

Based on Figure 1, the bioimpedance $Z_{m}(j\omega )$ was calculated as follows:

(1) $$Z_{m}\left(j\omega \right)=\frac{V_{2}}{V_{1}}\cdot R_{ref}=\frac{v_{2}}{v_{1}}e^{j\left(\phi_{2}-\phi_{1}\right)}\cdot R_{ref}$$

where $V_{2}=v_{2}e^{j\phi_{2}}$ and $V_{1}=v_{1}e^{j\phi_{1}}$ denote the measured potentials between the electrodes, while $R_{ref}=1000\;\Omega$ is the shunt resistance. In this setup, both the excitation and measurements are differential signals. The real and imaginary parts of the impedance were calculated using the following equations:

(2) $$R=\left|Z\right|\cos\;\left(\phi \right)$$

(3) $$X=\left|Z\right|\sin\;\left(\phi \right)$$

where R is the resistance (Ω), X is the reactance (Ω), ∣*Z*∣ is the magnitude of the impedance, and *ϕ* is the phase angle.

Healthy, disease-free leaves from different N treatments were selected for measurement, with each measurement repeated five times, resulting in a total of 60 measurements. BIS measurements were conducted on leaves at the same phenological level. To ensure high reproducibility, the leaves remained intact during the test. Four gold electrodes were positioned at equal intervals of 1.5 cm, following the methodology of [24] (Figure 1). To minimize contact impedance, a thin layer of ECG gel (Rextra ECG gel, Hungary) was applied at the interface between the leaf surface and each electrode. The electrodes were carefully placed to avoid contact with the leaf midrib, as described by [24]. After each measurement, the gold electrodes were thoroughly cleaned with deionized water to prevent contamination and were subsequently dried by wiping. The BIS measurements were conducted using a minimally invasive and non-destructive approach. Although the gold-coated needle electrodes penetrate the leaf surface, this process is considered minimally invasive because the electrodes cause only minor, localized damage to the leaf surface without significantly disrupting cellular functions or physiological processes. Additionally, the measurement process is regarded as non-destructive, as the leaf tissue remains alive and functional after measurement, with no substantial damage to the cells or overall leaf structure. Previous research [25] has demonstrated that this measurement technique does not affect cell viability, confirming the non-destructive properties of the method. This approach ensures that repeated measurements can be conducted on the same leaf without compromising the results.

In this study, the Double-Shell (DBS) model, as described by [26], was utilized for the cucumber ES2217 F1 genotype. The DBS model, based on the state-of-the-art literature, incorporates parameters such as extracellular fluid resistance ($R_{1}$), intracellular fluid resistance ($R_{2}$), vacuole resistance ($R_{4}$), and two non-ideal capacitive components corresponding to the cell membrane and vacuole membrane. Rather than modeling these components as pure capacitors, we employ constant phase elements (CPEs) to reflect the real, frequency-dependent properties of biological membranes [24,27,28,29,30].

**Figure 2:**
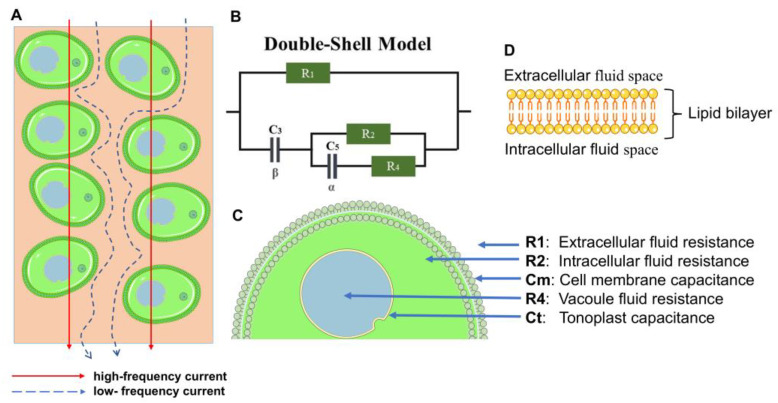
Overview of the Double-Shell model and its components. (**A**) Schematic representation of the current pathways within plant tissue, showing high-frequency current (red lines) and low-frequency current (blue dashed lines). (**B**) Equivalent electrical circuit of the DBS model, including extracellular fluid resistance ($R_{1}$), intracellular fluid resistance ($R_{2})$, vacuole resistance ($R_{4}$), cell membrane capacitance ($C_{m}$), and vacuole membrane capacitance ($C_{t}$), CPE characterizing the cell membrane ($C_{3}),\;$CPE characterizing the tonoplast ($C_{5}$), *α* associated with the vacuole membrane, and *β* associated with the cell membrane. (**C**) Cross-sectional diagram of a plant cell highlighting the parameters represented in the DBS model. (**D**) Structure of the lipid bilayer separating extracellular and intracellular fluid spaces, illustrating its role in electrical impedance.

Specifically, $C_{3}$ and $C_{5}$ in Figure 2 represent the CPEs for the cell membrane and tonoplast (vacuole membrane), respectively. The CPE exponents *β* (for $C_{3}$) and *α* (for $C_{5}$), both ranging between 0 and 1, quantify the degree of non-ideality [31]. A value of 1 indicates behavior closer to that of a purely capacitive (ideal) element, whereas values significantly below 1 signify broader distributions of relaxation times due to membrane heterogeneities.

By introducing these CPEs into the DBS equivalent circuit, we capture the non-ideal characteristics of biological membranes more accurately than an ideal capacitor model would. Consequently, the bioimpedance $Z\left(j\omega \right)$ of the DBS circuit can be written as follows [24]:

(4) $$\begin{array}{ccc} Z\left(j\omega \right)=\frac{R_{1}\left(\frac{1}{{\left(j\omega \right)}^{\beta }C_{3}}+Z_{partial}\right)}{R_{1}+\frac{1}{{\left(j\omega \right)}^{\beta }C_{3}}+Z_{partial}}, & where & Z_{partial}=\frac{R_{2}\left(R_{4}+\frac{1}{{\left(j\omega \right)}^{\alpha }C_{5}}\right)}{R_{2}+R_{4}+\frac{1}{{\left(j\omega \right)}^{\alpha }C_{5}}} \end{array}$$

In the aforementioned equation, ω denotes the angular frequency and $j=\sqrt{-1}$. Moreover, α∈(0,1) and β∈(0,1) characterize the CPE components, i.e., $Z_{CPE_{5}}=1/({\left(j\omega \right)}^{\alpha }C_{5})\;$represents the impedance of the $C_{5}$ capacitor, while $Z_{CPE_{3}}=1/({\left(j\omega \right)}^{\beta }C_{3})$ is the CPE-based relaxation of the impedance of the $C_{3}$ capacitor.

Although $C_{3}$ and $C_{5}$ appear formally as CPE parameters, we often present the resulting best-fit values as cell membrane capacitance ($C_{m}$) and vacuole membrane capacitance ($C_{t}$) for comparison with previous studies [12,14,16,17,24,32]. However, that these capacitances reflect the effective or apparent membrane properties under the non-ideal impedance behavior [31]. The time constants and other plant biological parameters derived from ($C_{m,\;}\;and\;C_{t}$) are calculated using Equations (5)–(8):

(5) $$\tau_{1}=\sqrt[{\alpha }]{R_{1}C_{3}}$$

(6) $$C_{m}=\frac{\tau_{1}}{R_{1}}$$

(7) $$\tau_{2}=\sqrt[{\beta }]{R_{2}C_{5}}$$

(8) $$C_{t}=\tau_{2}/R_{2}$$

Our previous [24] work demonstrated that these parameters ((5)–(8)) correlate strongly with important plant physiology parameters, such as relative water content, membrane stability index, and photosynthetic efficiency. This enhanced correlation suggests that the effective capacitances not only capture the non-ideal behavior of biological membranes, but also serve as more sensitive indicators of underlying cellular processes (e.g., membrane integrity loss under water stress). In this way, $C_{m,\;}$and $C_{t\;}$allow us to establish a more meaningful link between the electrical model and the physiological state of the plant tissues, thereby improving both the interpretability and the biological relevance of the BIS measurements.

In a previous paper, we developed a methodology for calibrating the complete measurement system using a physical model that mimics a DBS model, reconstructing the model parameters from the recorded impedance data. In this case, the parameters of the physical model and the reconstructed models were compared and the statistics performed indicated that the accuracy of the complete measurement system for the DBS model was at least 1%, regardless of the resolution of the measured spectra [24].

### 2.4. Numerical Optimization-Based Parameter Extraction

An optimization framework originally established in [24] was applied for parameter extraction. The cost function minimization process utilized a custom-built multi-objective fitness function F, which evaluated the differences in both magnitude and phase characteristics between the DS model and real spectrum measurements as the sum of the relative errors between the model-based estimated impedance and the measured impedance for each frequency point. The cost function applied for the optimization is detailed in Equation (9).

(9) $$F=\frac{1}{N}\sum_{i}^{N}\left|\frac{Z_{i}-Z_{m_{i}}}{Z_{m_{i}}}\right|+\frac{R_{1}}{10^{5}}+\frac{R_{2}}{10^{5}}+\frac{R_{4}}{10^{5}}+\frac{C_{3}}{10^{-6}}+\frac{C_{5}}{10^{-10}}+\alpha +\left|0.4-\beta \right|$$

where $Z_{i}$ and $Z_{m_{i}}$ denote the ith complex impedance values of the model and bioimpedance measurements, respectively. The running index i denotes the ith frequency component at which the measurement was executed, i.e., $Z_{i}=Z\left(j\omega_{i}\right)$ and $Z_{m_{i}}=Z_{m}\left(j\omega_{i}\right)$. N=50 is the total number of frequencies (in total, 10 frequencies were measurements in 5 decades). The aforementioned fitness function also included the scaled parameter values to suppress the parameter fluctuations. The derivation of this cost function is discussed in detail in [24]. The output of the optimization framework is the optimal model parameter set ($R_{1},R_{2},C_{3},R_{4},C_{5},\alpha$, and β), which belongs to the global minimum of F. The initial model parameters and the applied optimization ranges are summarized in Table 2, as described in [24].

The PSO algorithm was utilized in the optimization framework, the particle set of which was driven based on the evaluated fitness function F in each generation. This algorithm was proven to provide the most robust output in the parameter extraction process compared to the state-of-the-art solutions; see the results in [24]. The PSO was set up with the following hyperparameters: cognitive and social parameters—0.5 and 1.5, respectively; inertia weight—0.9; number of generations and populations—400 and 2000, respectively. This setup allowed for a well-distributed particle spread across the parameter space, ensured robust fitness evaluation against measurement noise, and facilitated convergence to the global optimum. The detailed description of this optimization framework setup is discussed in [24].

A total of 60 spectrum measurements (scenarios) were analyzed using the aforementioned optimization framework, with the optimal model parameters determined for each scenario by minimizing F.

**Figure 3:**
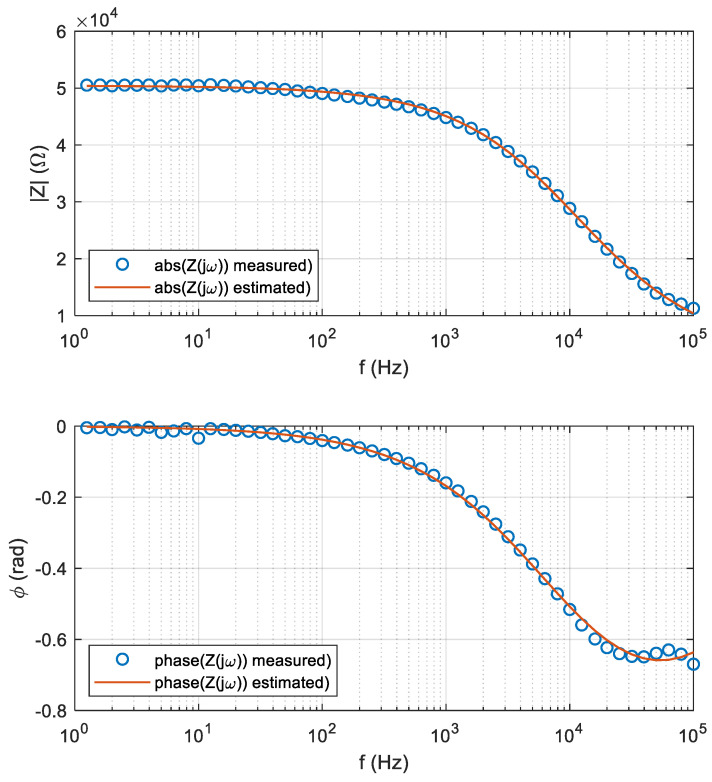
Spectra of both optimized models and measurements for a particular scenario.

**Figure 4:**
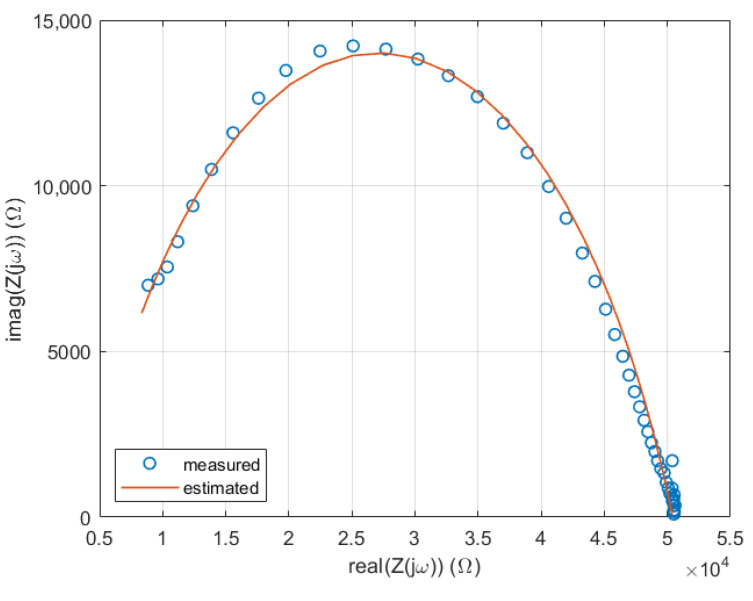
Cole–Cole diagrams of both optimized models and measurements for a particular scenario.

The following Figure 3 and Figure 4 show examples of the optimized BIS models (red curves) for a specific scenario (24/N3 treatment/cucumber leaves), along with the actual spectral measurements (blue dotted curves).

### 2.5. Statistical Analysis

The statistical analyses were performed utilizing R software (version 4.2.1). The impact of nitrogen treatments was analyzed through a MANOVA model, with the effectiveness of treatments assessed using Wilks’ λ statistic. Box’s test was employed to evaluate the homogeneity of the covariance matrix, while the Kolmogorov–Smirnov test was used to confirm the normality of residuals in the MANOVA for all dependent variables. The homogeneity of variance was tested using Levene’s test. Statistical significance was set at *p* < 0.05. The visualizations were generated using the ′ggplot2′ package, with the graphs displaying mean values alongside their corresponding standard deviations (±SD). The partial eta squared ($\eta_{p}^{2}$) was also calculated as an indicator of the MANOVA effect size [33].

(10) $$\eta_{p}^{2}=\frac{SS_{\mathrm{effect}}}{SS_{\mathrm{effect}}+SS_{\mathrm{error}}}$$

where $SS_{\mathrm{effect}}$ represents the sum of squares for the effect and $SS_{\mathrm{error}}\;$represents the sum of squares for the error. The relationships between the Cole–Cole parameters and plant nitrogen content were analyzed using Pearson correlation analysis. The Pearson correlation coefficient (r) measures the strength and direction of a linear relationship between two continuous variables. It is calculated as follows:

(11) $$r=\frac{\sum \left(X_{i}-\bar{X}\right)\left(Y_{i}-\bar{Y}\right)}{\sqrt{\sum {\left(X_{i}-\bar{X}\right)}^{2}}\sqrt{\sum {\left(Y_{i}-\bar{Y}\right)}^{2}}}$$

where $X_{i}$ and $Y_{i}\;$are individual data points, and $\bar{X}$ and $\bar{Y}$ are the mean values of the respective variables. The Pearson correlation coefficient (r) ranges from −1 to 1. r > 0 indicates a positive correlation, r < 0 indicates a negative correlation, and r = 0 suggests no linear relationship between the variables.

The optimal range of cell membrane capacitance, as well as intracellular and extracellular fluid resistance, was determined based on the mean (μ) and standard deviation (σ) values for each nitrogen treatment (N1, N2, N3, N4). In this experiment, N1 and N4 served as reference points to demarcate suboptimal ranges, while N2 and N3 were assumed to approximate balanced N availability. Specifically, the lower boundary of the optimal range was set to max (μN3 − σ N3, μN4 − σN4), thus ensuring that excessively low values characteristic of N4 conditions did not fall into the optimal domain. The upper boundary was determined as min (μN2 + σN2, μN3 + σN3), thereby excluding high values associated with lower N dose states. This statistical approach enabled us to identify ranges of capacitances and resistances that most consistently reflected balanced N supply, while minimizing the inclusion of extreme or biologically suboptimal measurements.

## 3. Results

### 3.1. Effect of N Treatments on Plant Physiology Parameters

The treatments significantly influenced the physiological parameters of the cucumber plants at the 6–7 true leaf phenological stage (F_(3,32)_ = 28.03; *p* < 0.001; Wilk’s λ = 0.27; partial $\eta_{p}^{2}$ = 0.72). The $\eta_{p}^{2}\;$value of 72% indicates that N treatments accounted for more than 70% of the variation in pigment content, as well as NO_3_^−^ and total N content. The lowest chlorophyll content was detected in N1 treatments, which was significantly different from the N2, N3, and N4 treatments (*p* < 0.05). No statistically significant difference was detected between the N3 and N4 treatments (*p* > 0.05). A similar trend was observed for the total N and NO_3_^−^ content of the cucumber leaves. The lowest leaf total N and NO_3_^−^ content was observed in N1 treatments, while the highest at higher N doses was observed in the N3 and N4 treatments (Table 3).

### 3.2. Effect of N Treatments on Bioimpedance Parameters

**Figure 5:**
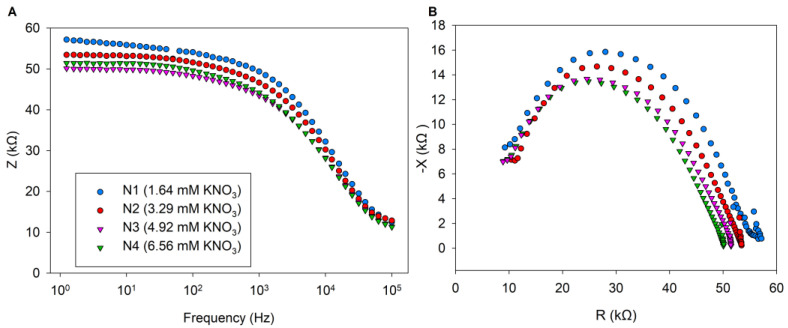
Changes in the bioimpedance spectroscopy of cucumber leaves as a dependence of frequency for samples with different N treatments. The plots represent the average impedance spectrum calculated from all measured samples within each N treatment (N1–N4). (**A**) Bode diagram representing the frequency dependence of impedance in cucumber leaves. (**B**) Cole–Cole plot illustrating the impedance characteristics of cucumber leaves.

At low frequencies, the impedance values decreased with increasing N dose. The N0 cucumber samples showed higher impedance values. In contrast, at higher N supply (N3–N4), the impedance values were reduced in cucumber plants (Figure 5A). The observed decrease in impedance values at low-frequency ranges is likely associated with changes in the extracellular fluid resistance. As the N dose increases between 4.92 mM and 6.56 mM, the impedance parameters at low frequency did not show remarkable separation. However, treatment with the lower concentration of 1.64 mM KNO_3_ results in a marked difference in the impedance parameters. The higher KNO_3_ treatments did not exhibit visually significant differences. The right and left sides of the Cole–Cole diagram (Figure 5B) represent different frequency ranges. The right side corresponds to low frequencies, while the left side reflects high frequencies. At high frequencies, the impedance values from the four treatments were very similar. However, at low frequencies, there were significant differences among the five treatments. Cucumber plants exposed to N1 and N2 treatments exhibited distinct impedance modulus values in the low-frequency range compared to those observed under other treatments. The lower impedance modulus in the other samples is associated with the higher total N and NO_3_^−^ content in the cucumber plants.

The treatments significantly influenced the BIS parameters of cucumber plants at the 6–7 true leaf phenological stage (F_(27,53)_ = 11.05; *p* < 0.001; Wilk’s λ= 0.004; $\eta_{p\;}^{2}$= 0.84). The Wilk’s λ value was smaller compared to that of the physiological parameters, indicating that the treatments had a stronger effect on the BIS parameters. Additionally, $\eta_{p}^{2}\;$exceeded 84%, highlighting the significant impact of the treatments on these (Cole–Cole) parameters.

**Figure 6:**
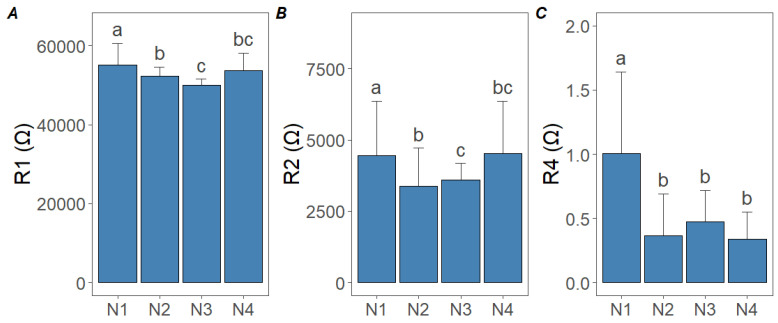
Results of the Double-Shell model with PSO optimization algorithm for the average of all four treatments on cucumber plants. (**A**) Extracellular fluid resistance ($R_{1}$ ); (**B**) intracellular fluid resistance ($R_{2})$; (**C**) vacuole resistance ($R_{4}$). Values are means ± standard deviation (n = 4). Lowercase letters indicate significant differences between treatments according to Tukey’s HSD, *p* < 0.05.

Significant differences were observed in the impedance parameters ($R_{1}$, $R_{2}$, and $R_{4}$) across the treatments. For $R_{1}\;$resistance, the highest values were found in the N1 and N2 samples, with a decreasing trend as the N doses increased, followed by a slight increase in the N4 treatment (Figure 6A). For $R_{1}$ resistance, $\eta_{p}^{2}\;$exceeded 0.90. Similarly, $R_{2}$ resistance was highest in the N1 treatment, progressively decreasing with higher N doses, reaching its lowest value in N3, and slightly increasing again in N4 (Figure 6B). For $R_{2}$, resistance was lower ($\eta_{p\;}^{2}$ = 0.67) than $R_{1}$ resistance. In contrast, $R_{4}\;$resistance showed a significant decrease with increasing N doses, where N1 exhibited the highest resistance, while the N2, N3, and N4 treatments maintained consistently low values without significant differences (Figure 6C). These results suggest that N1 and N2 treatments increase resistance values, while higher doses cause a reduction, with minor variability observed among the N3 and N4 treatments. Regarding $R_{4}$ resistance, $\eta_{p}^{2}\;$did not exceed 0.34.

In the N1 and N2 treatments a decrease in the $R_{1}$/$R_{2\;}$ratio was observed compared to the other treatments, which may indicate changes in the cell membrane. The average $R_{1}$/$R_{2\;}$ratio for the N1 treatment was significantly lower than the other treatments (*p* < 0.05), by 54.96% compared to N2, 44.22% compared to N3, and 40.92% compared to N4.

**Figure 7:**
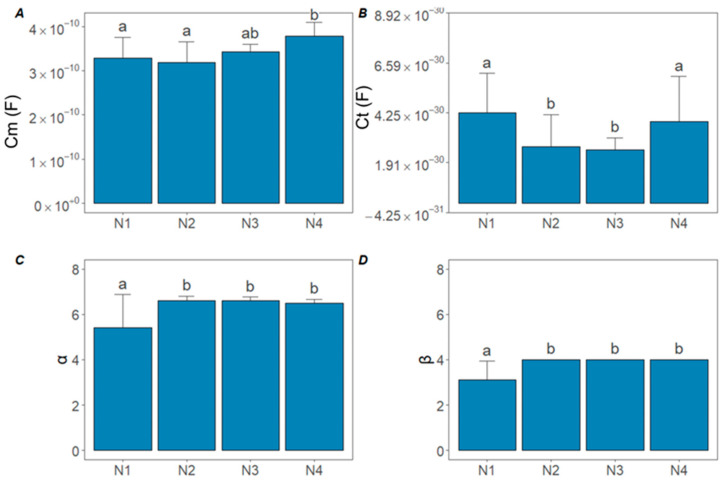
Results of the Double-Shell model with PSO optimization algorithm for the average of all four treatments on cucumber plants. (**A**) Cell membrane capacitance ($C_{m}$ ); (**B**) vacuole membrane capacitance ($C_{t}$); (**C**) α associated with the vacuole membrane; (**D**) β associated with the cell membrane. Lowercase letters indicate significant differences between treatments according to Tukey’s HSD, *p* < 0.05.

As observed with the significantly lower $R_{1}$/$R_{2}$ ratio in the N1 samples, the $C_{m}$ capacitance values were also reduced in the N1 and N2 samples. In contrast, higher $C_{m}$ capacitance values were recorded in samples with higher N content (Figure 7A). Regarding $C_{t}$ capacitance, no significant difference was detected between the N1 and N2 samples (*p* > 0.05) (Figure 7B). Regarding the capacitances ($C_{m},C_{t}$) $\eta_{p}^{2}\;$did not exceed 0.34. For the α and β parameters, only the N1 sample showed a significant difference (*p* < 0.05) (Figure 7C,D).

**Figure 8:**
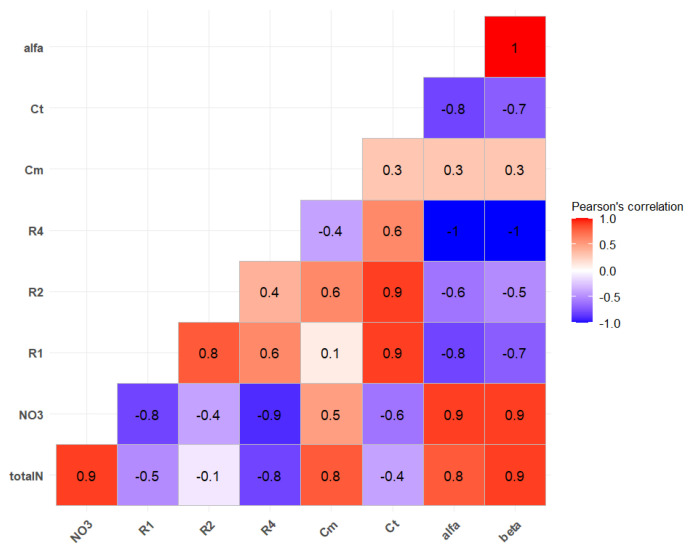
Pearson correlation matrix between bioimpedance and nitrogen supply parameters. (Note: $R_{1}$ : extracellular fluid resistance; $R_{2}$: intercellular fluid resistance; $R_{4}:\;$vacuole fluid resistance; $C_{m}:$ plasma membrane capacitance; $C_{t}:$ vacuole capacitance; α: associated with the cell membrane; β associated with the vacuole membrane; total N: Total N m/m %; NO_3_: NO_3_^−^ mg g^−1^ FW.)

The correlation analysis shows that the impedance parameters are closely related to the N and NO_3_^−^ content: a strong positive correlation between total N and NO_3_^−^ is observed (r = 0.9), while there is a strong negative correlation between NO_3_^−^ content and $R_{1}$ (r = −0.8). Increases in NO_3_^−^ levels are also associated with decreases in intracellular $R_{2}$ and vacuole fluid resistance $R_{4}$ (r = −0.4 and r = −0.9) (Figure 8). Based on these findings, the optimal N supply in cucumber plants is characterized by the following bioimpedance parameter ranges: $R_{1}$ between 47,121.07 and 52,953.93 Ω; vacuole fluid resistance $R_{4}$ between 0.348 and 0.529 Ω; and $C_{m}\;$between 3.149 × 10⁻^10^ and 3.781 × 10⁻^10^ F.

## 4. Discussion

Cucumber samples from the N1 and N2 treatments exhibited higher impedance values, whereas those from the N3 and N4 treatments displayed lower impedance values (Figure 5A,B). This is related to the NO_3_^−^ and total N content of the cucumber plants (Table 1). The impedance results were obtained by the authors of [14], who also observed that tomato leaves from samples with lower total N content showed higher impedance values, which can be explained by the fact that leaf samples with low total N concentration also have low NO_3_^−^ ion concentration, and therefore a high impedance modulus correlates with low total N concentration. Similarly, in previous studies, higher impedance values have been observed for samples with low phosphorus and potassium content in the leaves [16,17]. However, in our case, the main differences could only be due to the N treatments, as the other nutrients were provided in optimal amounts. The impedance values of the N3 and N4 samples did not differ remarkably in different frequency bands (Figure 5A,B), and the total N and NO_3_^−^ content of cucumber leaves did not differ statistically significantly for higher N3–N4 doses (Table 2). Similarly, the resistances ($R_{1}$, $R_{2}$, $R_{4}$) and capacitances ($C_{t}$, $C_{m}$) showed no significant differences between the N3 and N4 treatments (Figure 6 and Figure 7). This may be explained by the tight regulation of NO_3_^−^ uptake through the feedback suppression of transport [21,34], which could be attributed to increased amino acid levels resulting in decreased NO_3_^−^ uptake. However, further studies are required to confirm this finding. In the single Cole–Cole model, bioimpedance measurements are used to characterize the extracellular and intracellular fluid resistance properties of biological tissues [35]. In our study, extracellular resistance reached its highest value in the N1 treatment (55,037.5 Ω), whereas the N3 treatment produced the lowest extracellular resistance (50,037.5 Ω). In cucumber plants with low total N and NO_3_^−^ content, higher extracellular fluid resistance was observed, whereas plants with higher total N and NO_3_^−^ content exhibited lower resistance values (Figure 6A). The increase in extracellular resistance in N1 and N2 treatments can be attributed to differences in NO_3_^−^ and NH_4_^+^ ion concentrations [14]. Since cucumber plants with low N supply had lower total N and NO_3_^−^ content (Table 2), the increase in extracellular fluid resistance is likely explained by reduced NO_3_^−^ saturation in the extracellular fluid space after NO_3_^−^ uptake [36], altering the resistance of the extracellular fluid (Figure 6A). This phenomenon can be explained by the fact that biological tissues consist of a complex network of cells, extracellular matrix, macromolecules, small molecules, ions, and water. Both extracellular and intracellular fluids can be considered as liquid electrolytes, which vary in viscosity with temperature, thus affecting ion mobility and consequently resistance [9,14,37]. At low frequencies, current primarily flows through the extracellular fluid (Figure 2A), and since extracellular resistance increased in low N supply, low-NO_3_^−^ ions can be detected in this frequency range.

Concerning the intracellular fluid resistance, the highest value was observed in N1 cucumber plants (≈4451.25 Ω), indicating that low N availability might reduce the ionic pool or mobility in the cytoplasm, thereby increasing its resistance. In contrast, moderate or high N levels (N2, N3, N4) provided more favorable ionic levels, keeping intracellular fluid resistance at relatively lower levels.

Vacuole resistance exhibited a similar trend, with N1 showing the highest value (1.007 Ω) and N2–N4 falling in a lower range (0.339–0.472 Ω). Low NO_3_^−^ samples showed significantly higher vacuole resistance values, likely because vacuoles, as storage pools, play a critical role in NO_3_^−^ storage [38,39,40]. However, it is important to consider the work of [27] Nouaze et al. (2022), who observed that vacuole fluid resistance may only change slightly, as vacuoles are composed of approximately 90% water, and ion transfer from vacuoles to the cytoplasm and cell walls during growth minimally affects their absolute ion concentration. Additionally, differences in certain leaf morphological traits, including xylem N content, may also contribute to variations [40].

Collectively, these findings confirm the hypothesis that variations in N supply alter the ion balance in both extracellular and intracellular compartments, thereby modulating the bioimpedance signatures ($R_{1}$, $R_{2}$, $R_{4}$) of cucumber leaves. The cell membrane capacitance and vacuole capacitance showed distinct values among the cucumber samples with different N levels (Figure 7A,B). Cell membranes separate the intracellular fluid from the extracellular fluid, making a barrier against the free movement of ions and large molecules. They consist of a lipid bilayer associated with proteins, ion channels, and pumps that ensure the active functions of the membrane. The membrane itself is a poor electrical conductor and behaves as a dielectric (Figure 2D) [37]. A reduction in cell membrane capacitance was observed in cucumber plants with low N levels (Figure 7A). This can be attributed to the fact that the low N supply affects membrane stability and fluidity [41], thereby reducing the charge storage capacity of the membrane surface. Low N supply was characterized by lower α and β parameters. The α parameter is associated with the vacuole membrane, while β corresponds to the cell membrane [12]. Moreover, the primary counter ion for nitrate transport is K^+^ [34], whose availability may also influence membrane capacitance, but further research is required to clarify these details.

Meanwhile, low N supply levels influenced membrane stability and fluidity, leading to reduced structural integrity and charge storage capacity in both the vacuole membrane (α) and cell membrane (β). This indicates that lower N supply affects the functional capacity of membranes, such as ion transport and osmoregulation, which can affect the physiological processes within cells. However, deeper molecular studies are needed to fully understand these effects.

Here, we propose using plant leaves for impedance measurements to detect N treatments in cucumber plants. The suggested BIS measurement setup, together with gold electrodes, exhibited strong performance and provided high-quality data for extracting information on N content. The results obtained validate both the proposed measurement method and the optimization approach based on the Double-Shell model, thereby indicating that the recommended inference approach could be a promising new technology for analyzing plant structures. It is important to note that BIS measurements can be sensitive to various external and internal factors [8,10,11,14,16,17,27,42]. In this study, we minimized such variability by standardizing measurement conditions, including temperature, sample handling, and electrode placement, as well as taking into account leaf morphology and hydration status. Another critical point is that ions other than NO_3_^−^, such as K^+^, Ca^2+^, Mg^2+^, and phosphate, can alter the electrochemical environment and potentially interfere with the measured spectra [16,17]. Although our nutrient solutions are designed to maintain these ions at optimal concentrations, minor variations in uptake or internal redistribution cannot be completely excluded. Changes in the quantity of individual ions can shift the relative contributions of extracellular and intracellular compartments, thereby affecting both resistance and capacitance values [14,16,17,43]. Further small pot experiments, possibly involving multi-element analyses or tests with different ions, could provide deeper insights into how these ions individually and collectively modify impedance values. Such studies may clarify whether certain ion profiles can mask or amplify the effects attributed to NO_3_^−^ availability, particularly for K^+^, given its role as a NO_3_^−^ counterion [44]. Despite these challenges, the consistency of our present results indicates that the dominant factor influencing the BIS of the cucumber leaves studied here was N availability. Nevertheless, carefully designed future studies are warranted to assess and mitigate the effects of interfering ions in a systems approach and to confirm the reproducibility of BIS measurements in plant tissues.

## 5. Conclusions and Future Work

Our results confirm that bioimpedance spectroscopy (BIS) can be used as a rapid, non-destructive tool to assess the N supply of cucumber plants grown in greenhouses and can help to make fertilization decisions that affect yield and crop quality. Specifically, we found the following:

(1) BIS parameters, particularly extracellular fluid resistance and cell membrane capacitance, showed clear, statistically significant responses to different N doses. These changes reflect physiological processes such as nitrate saturation in the apoplast and membrane stability.
(2) Although increasing N doses generally lowered resistance and decreased cell membrane capacitance, the total N and NO_3_^−^ contents did not continue to rise indefinitely, indicating feedback suppression in NO_3_^−^ uptake. This threshold effect highlights the importance of precise N management to avoid wasteful over-fertilization without gaining additional yield or quality benefits.
(3) By detecting early signs of low or excess N supply, BIS measurements can complement traditional methods (e.g., Kjeldahl or NO_3_^−^ assays) and help growers optimize fertilizer application.
(4) While the current work focused on the 6–7 true leaf stage, future studies could map BIS responses across multiple growth phases and correlate them directly with yield metrics (e.g., fruit number, size, quality parameters). Additionally, integrating BIS data with other non-destructive diagnostic tools (e.g., hyperspectral imaging) could further refine real-time monitoring and fertilization strategies under commercial greenhouse conditions.
(5) Future work will also include a more detailed investigation of the biological meaning of the α and β parameters. In our future studies, we plan to analyze these parameters more thoroughly to understand how they relate to cell membrane dynamics and other physiological processes. This deeper insight will allow us to further refine our BIS models and improve the interpretation of plant nutrient status and stress responses.

## Figures and Tables

**Table 2:** Parameters, initialization, and ranges of the optimization problem.

Parameter	Initial Value	Range
$R_{1}\left(\Omega \right)$	$5\cdot 10^{6}$	$\left[1\cdot 10^{-3},10\cdot 10^{6}\right]$
$R_{2}\left(\Omega \right)$	$5\cdot 10^{6}$	$\left[1\cdot 10^{-3},10\cdot 10^{6}\right]$
$C_{3}\left(\frac{F}{s^{1-\beta }}\right)$	$15\cdot 10^{-6}$	$\left[1\cdot 10^{-14},1\cdot 10^{-3}\right]$
$R_{4}\left(\Omega \right)$	$5\cdot 10^{6}$	$\left[1\cdot 10^{-3},10\cdot 10^{6}\right]$
$C_{5}\left(\frac{F}{s^{1-\alpha }}\right)$	$15\cdot 10^{-6}$	$\left[1\cdot 10^{-14},1\cdot 10^{-3}\right]$
α	0.5	$\left[1\cdot 10^{-3},1.0\right]$
β	0.5	$\left[1\cdot 10^{-3},1.0\right]$

**Table 3:** Summary of ANOVA results for physiological parameters of cucumber plants under different nitrogen treatments (N1–N4). Values are means ± standard deviation (n = 4). Letters indicate significant differences among treatments (Tukey’s HSD, *p* < 0.05).

Treatment	CCI	Total N m/m %	NO_3_^−^ mg g^−1^ FW	Chla-a mg g^−1^ FW	Chla-b mg g^−1^ FW	Total-chl mg g^−1^ FW	Carotenoids mg g^−1^ FW
N1	37.93 ± 0.86 ^a^	4.41 ± 0.07 ^a^	0.37 ± 0.17 ^a^	3.56 ± 0.07 ^a^	1.15 ± 0.02 ^a^	4.55 ± 0.09 ^a^	0.63 ± 0.01 ^a^
N2	43.33 ± 1.80 ^b^	4.87 ± 0.15 ^b^	0.43 ± 0.36 ^b^	4.05 ± 0.16 ^b^	1.31 ± 0.05 ^b^	5.16 ± 0.20 ^b^	0.71 ± 0.02 ^b^
N3	47.33 ± 3.17 ^c^	5.22 ± 0.27 ^c^	0.47 ± 0.64 ^c^	4.41 ± 0.28 ^c^	1.43 ± 0.09 ^bc^	5.62 ± 0.36 ^c^	0.77 ± 0.04 ^c^
N4	49.70 ± 4.46 ^c^	5.42 ± 0.38 ^c^	0.46 ± 0.89 ^c^	4.63 ± 0.40 ^c^	1.49 ± 0.12 ^c^	5.89 ± 0.50 ^d^	0.80 ± 0.06 ^c^
F values	28.03	25.03	22.03	28.03	26.03	28.03	25.03
$\eta_{p}^{2}$	0.72	0.71	0.69	0.72	0.60	0.72	0.52
*p* values	<0.001	<0.001	<0.001	<0.001	<0.001	<0.001	<0.05

## Data Availability

The data presented in this study are available on request from the corresponding author.
